# Higher CSF sTREM2 attenuates ApoE4-related risk for cognitive decline and neurodegeneration

**DOI:** 10.1186/s13024-020-00407-2

**Published:** 2020-10-08

**Authors:** Nicolai Franzmeier, M. Suárez-Calvet, Lukas Frontzkowski, Annah Moore, Timothy J. Hohman, Estrella Morenas-Rodriguez, Brigitte Nuscher, Leslie Shaw, John Q. Trojanowski, Martin Dichgans, Gernot Kleinberger, Christian Haass, Michael Ewers, for the Alzheimer’s Disease Neuroimaging Initiative (ADNI), Michael Weiner, Paul Aisen, Gerald Novak, Robert C. Green, Tom Montine, Ronald Petersen, Anthony Gamst, Ronald G. Thomas, Michael Donohue, Sarah Walter, Devon Gessert, Tamie Sather, Laurel Beckett, Danielle Harvey, John Kornak, Clifford R. Jack, Anders Dale, Matthew Bernstein, Joel Felmlee, Nick Fox, Paul Thompson, Norbert Schuff, Gene Alexander, Charles DeCarli, William Jagust, Dan Bandy, Robert A. Koeppe, Norm Foster, Eric M. Reiman, Kewei Chen, Chet Mathis, John Morris, Nigel J. Cairns, Lisa Taylor-Reinwald, J. Q. Trojanowki, Les Shaw, Virginia M. Y. Lee, Magdalena Korecka, Arthur W. Toga, Karen Crawford, Scott Neu, Andrew J. Saykin, Tatiana M. Foroud, Steven Potkin, Li Shen, Zaven Kachaturian, Richard Frank, Peter J. Snyder, Susan Molchan, Jeffrey Kaye, Sara Dolen, Joseph Quinn, Lon S. Schneider, Sonia Pawluczyk, Bryan M. Spann, James Brewer, Helen Vanderswag, Judith L. Heidebrink, Joanne L. Lord, Kris Johnson, Rachelle S. Doody, Javier Villanueva-Meyer, Munir Chowdhury, Yaakov Stern, Lawrence S. Honig, Karen L. Bell, John C. Morris, Mark A. Mintun, Stacy Schneider, Daniel Marson, Randall Griffith, David Clark, Hillel Grossman, Effie Mitsis, Aliza Romirowsky, Leyla deToledo-Morrell, Raj C. Shah, Ranjan Duara, Daniel Varon, Peggy Roberts, Marilyn Albert, Chiadi Onyike, Stephanie Kielb, Henry Rusinek, Mony J. de Leon, Lidia Glodzik, P. Murali Doraiswamy, Jeffrey R. Petrella, Steven E. Arnold, Jason H. Karlawish, David Wolk, Charles D. Smith, Greg Jicha, Peter Hardy, Oscar L. Lopez, Mary Ann Oakley, Donna M. Simpson, M. Saleem Ismail, Connie Brand, Ruth A. Mulnard, Gaby Thai, Catherine Mc-Adams-Ortiz, Ramon Diaz-Arrastia, Kristen Martin-Cook, Michael DeVous, Allan I. Levey, James J. Lah, Janet S. Cellar, Jeffrey M. Burns, Heather S. Anderson, Russell H. Swerdlow, George Bartzokis, Daniel H. S. Silverman, Po H. Lu, Liana Apostolova, Neill R. Graff-Radford, Francine Parfitt, Heather Johnson, Martin Farlow, Scott Herring, Ann M. Hake, Christopher H. van Dyck, Richard E. Carson, Martha G. MacAvoy, Howard Chertkow, Howard Bergman, Chris Hosein, Sandra Black, Bojana Stefanovic, Curtis Caldwell, Ging-Yuek Robin Hsiung, Howard Feldman, Michele Assaly, Andrew Kertesz, John Rogers, Dick Trost, Charles Bernick, Donna Munic, Chuang-Kuo Wu, Nancy Johnson, Marsel Mesulam, Carl Sadowsky, Walter Martinez, Teresa Villena, Raymond Scott Turner, Kathleen Johnson, Brigid Reynolds, Reisa A. Sperling, Meghan Frey, Keith A. Johnson, Allyson Rosen, Jared Tinklenberg, Wes Ashford, Marwan Sabbagh, Christine Belden, Sandra Jacobson, Ronald Killiany, Alexander Norbash, Anil Nair, Thomas O. Obisesan, Saba Wolday, Salome K. Bwayo, Alan Lerner, Leon Hudson, Paula Ogrocki, Evan Fletcher, Owen Carmichael, Smita Kittur, Michael Borrie, T-Y Lee, Rob Bartha, Sterling Johnson, Sanjay Asthana, Cynthia M. Carlsson, Steven G. Potkin, Adrian Preda, Dana Nguyen, Pierre Tariot, Adam Fleisher, Stephanie Reeder, Vernice Bates, Horacio Capote, Michelle Rainka, Barry A. Hendin, Douglas W. Scharre, Maria Kataki, Earl A. Zimmerman, Dzintra Celmins, Alice D. Brown, Hartford Hosp, Godfrey D. Pearlson, Karen Blank, Karen Anderson, Robert B. Santulli, Eben S. Schwartz, Jeff D. Williamson, Kaycee M. Sink, Franklin Watkins, Brian R. Ott, Henry Querfurth, Geoffrey Tremont, Stephen Salloway, Paul Malloy, Stephen Correia, Howard J. Rosen, Bruce L. Miller, Jacobo Mintzer, Crystal Flynn Longmire, Kenneth Spicer

**Affiliations:** 1 Institute for Stroke and Dementia Research (ISD), University Hospital, Ludwig Maximilian University (LMU), Munich, Germany; 2 Barcelonaβeta Brain Research Center (BBRC), Pasqual Maragall Foundation, Barcelona, Spain; 3 IMIM (Hospital del Mar Medical Research Institute), Barcelona, Spain; 4 Servei de Neurologia, Hospital del Mar, Barcelona, Spain; 5 Vanderbilt Memory & Alzheimer’s Center, Vanderbilt University Medical Center, Nashville, USA; 6 Chair of Metabolic Biochemistry, Biomedical Center (BMC), Faculty of Medicine, Ludwig-Maximilians-Universität München, Munich, Germany; 7 Center for Neurodegenerative Disease Research, Institute on Aging, Perelman School of Medicine University, Philadelphia, USA; 8 Department of Pathology and Laboratory Medicine, Perelman School of Medicine, University of Pennsylvania, Philadelphia, PA USA; 9 Munich Cluster for Systems Neurology, Munich, Germany; 10 German Center for Neurodegenerative Diseases (DZNE), Munich, Germany; 11 ISAR Bioscience, Planegg, Germany

**Keywords:** Alzheimer’s disease, ApoE4, Microglial activation, sTREM2, Cognitive decline, Neurodegeneration

## Abstract

**Background:**

The Apolipoprotein E ε4 allele (i.e. ApoE4) is the strongest genetic risk factor for sporadic Alzheimer’s disease (AD). TREM2 (i.e. Triggering receptor expressed on myeloid cells 2) is a microglial transmembrane protein brain that plays a central role in microglia activation in response to AD brain pathologies. Whether higher TREM2-related microglia activity modulates the risk to develop clinical AD is an open question. Thus, the aim of the current study was to assess whether higher sTREM2 attenuates the effects of ApoE4-effects on future cognitive decline and neurodegeneration.

**Methods:**

We included 708 subjects ranging from cognitively normal (CN, *n* = 221) to mild cognitive impairment (MCI, *n* = 414) and AD dementia (*n* = 73) from the Alzheimer’s disease Neuroimaging Initiative. We used linear regression to test the interaction between ApoE4-carriage by CSF-assessed sTREM2 levels as a predictor of longitudinally assessed cognitive decline and MRI-assessed changes in hippocampal volume changes (mean follow-up of 4 years, range of 1.7-7 years).

**Results:**

Across the entire sample, we found that higher CSF sTREM2 at baseline was associated with attenuated effects of ApoE4-carriage (i.e. sTREM2 x ApoE4 interaction) on longitudinal global cognitive (*p* = 0.001, Cohen’s *f*^*2*^ = 0.137) and memory decline (*p* = 0.006, Cohen’s *f*^*2*^ = 0.104) as well as longitudinally assessed hippocampal atrophy (*p* = 0.046, Cohen’s *f*^*2*^ = 0.089), independent of CSF markers of primary AD pathology (i.e. Aβ_1–42_, p-tau_181_). While overall effects of sTREM2 were small, exploratory subanalyses stratified by diagnostic groups showed that beneficial effects of sTREM2 were pronounced in the MCI group.

**Conclusion:**

Our results suggest that a higher CSF sTREM2 levels are associated with attenuated ApoE4-related risk for future cognitive decline and AD-typical neurodegeneration. These findings provide further evidence that TREM2 may be protective against the development of AD.

## Background

The *APOE* ε4 allele (i.e. ApoE4) is the strongest genetic risk factor for sporadic Alzheimer’s disease (AD) [1]. ApoE4-homozygotes have a 31–40% lifetime risk for developing AD dementia [2] and show ~ 10-year earlier AD symptom onset than ApoE4 non-carriers [3]. ApoE4-carriage is associated with AD-typical alterations in cerebrospinal-fluid (CSF) Aβ_1–42_ [4] and with earlier and stronger PET-assessed amyloid-beta (Aβ) [5, 6] and tau accumulation [7]. Additionally, elderly ApoE4-carriers show faster cognitive decline than ApoE4-non carriers [8–11] as well as AD-typical temporo-parietal neurodegeneration [12]. Together, ApoE4-carriers are at increased risk of developing primary AD pathology, cognitive decline and AD dementia [2, 5, 6]. Recent GWAS suggest that the brain’s immune response may modulate AD risk [13–15]. This is supported by a 2–4-fold elevated odds ratio for AD in carriers of loss-of-function risk variants in the *TREM2* gene (triggering receptor expressed on myeloid cells 2, i.e. in the brain preferentially expressed on microglia) [16]. The TREM2/DAP12 signaling complex regulates microglial responses to pathogens, enhancing microglia motility, chemotaxis and phagocytosis [17–22]. In APP or Tau transgenic mice, TREM2-mediated microglia activation has been shown to promote Aβ phagocytosis [20], to limit Aβ seeding [22], tau hyperphosphorylation [23] and tau seeding around neuritic plaques [24]. TREM2-deficiency has been associated with memory impairment in tau transgenic mice, while higher TREM2 expression was neuroprotective and beneficial for memory [25]. Similarly, enhancing TREM2 signaling via agonistic antibodies can promote microglial survival and Aβ phagocytosis in Aβ mice [26]. Further supporting protective TREM2 effects in AD, TREM2-deficiency is associated with reduced microglia clustering and increased tau seeding around neuritic plaques [24]. Hence, TREM2-related microglial activation may attenuate downstream consequences of primary AD pathology and modulate ApoE4-related risk for AD symptoms and neurodegeneration.

Soluble TREM2 (sTREM2) originates from ADAM10/17-mediated TREM2-ectodomain shedding and can be detected in the CSF [19, 27–29], hence sTREM2 levels are interpreted as proxies of microglial TREM2 signaling [27–29]. Supporting this, brain sTREM2 levels are highly correlated with TSPO-PET-assessed microglial activation in mice [30] and are decreased in mice carrying the TREM2 p.T66M variant that typically locks microglia in a homeostatic state [31]. We previously found in sporadic and familial AD that CSF sTREM2 levels rise before symptom onset and mostly correlate with increasing CSF tau levels, suggesting an adaptive immune response [32, 33]. Importantly, *higher* CSF sTREM2 levels at a given level of primary AD pathology (measured as the CSF Aβ_1–42_ and p-tau_181_), were associated with attenuated future cognitive decline, neurodegeneration and delayed conversion to AD dementia in amyloid-positive individuals [34]. These findings suggested that TREM2-related microglial activation is associated with attenuated cognitive decline and neurodegeneration in AD, especially when TREM2 levels are high at a given level of tau pathology.

Here, we asked whether CSF sTREM2 attenuates genetic risk for i) cognitive decline and ii) AD-typical neurodegeneration as conferred by the most important genetic AD risk factor ApoE4. This question is clinically important, since TREM2-signalling is modifiable [26] and may constitute a target for AD prevention. In a large sample of 708 subjects ranging from cognitively normal to dementia, we thus determined whether higher CSF sTREM2 levels attenuate the association between ApoE4-carriage and future cognitive decline or MRI-assessed neurodegeneration.

## Methods

### Sample

We included 708 subjects from the ADNI database with available ApoE4 genotyping, baseline CSF values of Aβ_1–42_, p-tau_181_ and sTREM2 as well as at least 1.5 years of clinical follow-up assessment (i.e. baseline plus two follow-up visits). ApoE4 genotyping methods can be found online (http://adni.loni.usc.edu/methods/). Subjects were characterized as ApoE4 carriers, when carrying at least one ApoE4 allele. Selection bias was tested against the ADNI baseline cohort of 1784 subjects. Here, we found no differences in gender or education between our selected sample and the baseline ADNI cohort, but selected subjects were significantly older (*p* < 0.05) than the entire ADNI cohort. Subjects were clinically classified by ADNI centers as cognitively normal (CN, MMSE> 24, CDR = 0, non-depressed), mild cognitively impaired (MCI; MMSE> 24, CDR = 0.5, objective memory-loss on the education adjusted Wechsler Memory Scale II, preserved activities of daily living) [35] or AD dementia following-pre-established criteria [35]. Based on pre-established CSF Aβ_1–42_ cut-offs at 976.6 pg/ml, subjects were classified as Aβ + (i.e. below 976.6 pg/ml) or Aβ- (i.e. above 976.6 pg/ml) [36]. ADNI was ethically approved by the institutional review board of all participating sites, subjects provided written informed consent.

### CSF biomarker assessment

CSF sTREM2 levels were measured using a previously described ELISA approach [19, 27]. Detailed methods of the CSF sTREM2 assessments can be found online in the ADNI LONI Image & Data Archive (https://ida.loni.usc.edu). CSF Aβ_1–42_ and p-tau_181_ levels were measured by the ADNI biomarker core at the University of Pennsylvania, using the electrochemiluminiscence immunoassays Elecsys on a fully automated Elecsys cobas e 601 instrument and a single lot of reagents for each biomarker.

### Hippocampal volume and cortical thickness assessment

Hippocampal volume was assessed longitudinally in 558 of the 708 subjects who had ≥3 available 3 T MRI-assessments provided by the ADNI imaging core at UCSF [37]. Hippocampal volumes were assessed on 3 T structural MRI (MPRAGE) using established FreeSurfer pipelines (Version 5.1). Protocols of the ADNI FreeSurfer-based pipelines are available online (http://adni.loni.usc.edu/) and in previous publications [38]. All analyses using hippocampal volumes as a dependent variable were controlled for Freesurfer-assessed intracranial volume.

### Cognitive assessment

Memory performance was determined using ADNI-MEM, a composite memory score that summarizes multiple tests including the Rey Auditory Verbal Learning Test, AD Assessment Scale – Cognitive Subscale, Word Recall of the MMSE and the Wechsler Logical Memory Scale II [39]. For global cognition, we used the ADAS13 [40, 41], which is frequently used as a primary endpoint in clinical trials. Please note that ADNI-MEM and ADAS13 scores have an inverse relationship, i.e. lower ADNI-MEM and higher ADAS13 scores indicate worse cognitive performance.

### Statistical analysis

Baseline characteristics were compared between diagnostic groups using ANOVAs for continuous and χ2-tests for categorical measures. To validate ApoE4 as a major AD risk factor in the current sample, we tested whether ApoE4-carriage is associated with more abnormal CSF AD biomarkers (i.e. CSF Aβ_1–42_ & p-tau_181_), using ANCOVAs, controlling for age, gender, education and diagnosis. We used equivalent ANCOVA models to assess whether ApoE4-carriage was associated with elevated CSF sTREM2 levels.

Next, we tested whether ApoE4-carriage was associated with faster rates of longitudinally assessed cognitive decline and neurodegeneration and whether higher sTREM2 levels moderated this association. As measures of cognition, we used ADNI-MEM and ADAS13. As a measure of neurodegeneration, we used Freesurfer-derived hippocampal volumes. To determine annual change rates in cognition and hippocampal volume, we employed a pre-established approach [42] in which we fitted linear mixed models with ADNI-MEM, ADAS13, hippocampal volumes or cortical thickness values as the dependent variable and time (i.e. years from baseline) as the independent variable, controlling for random slope and intercept. From the linear mixed models, we then derived a slope estimate for change in ADAS13, ADNI-MEM or hippocampal volume across time (i.e. change per year) for each subject. For each measure, longitudinal analyses were restricted to subjects with at least 3 available timepoints (i.e. *n* = 708 for ADNI-MEM and ADAS13; *n* = 558 for hippocampal volume). Using ANCOVAs, we tested whether ApoE4-carriage was associated with faster annual change rates in ADNI-MEM, ADAS13, hippocampal volume. These analyses were controlled for age, gender, education, diagnosis, follow-up time, baseline values of the dependent variable and intracranial volume when using hippocampal volume as a dependent variable.

For our main hypothesis, we assessed whether higher sTREM2 attenuates the association between ApoE4-carriage and cognitive decline as well as hippocampal volume changes. First, we applied three ANCOVA models, testing the interaction sTREM2 x ApoE4-carriage on annual rates of change in ADNI-MEM, ADAS13 or hippocampal volume. When using ADNI-MEM or ADAS13 change rates as dependent variables, models were controlled for main effects of sTREM2, ApoE4, p-tau_181_, Aβ_1–42_, age, gender, education, diagnosis, follow-up time and baseline cognition (i.e. ADNI-MEM or ADAS13). When using hippocampal volume change rates as the dependent variable, the model was controlled for main effects of sTREM2, ApoE4, Aβ_1–42_, p-tau_181_, age, gender, education, diagnosis, follow-up time, as well as baseline hippocampal volume and intracranial volume. These covariates were selected to ensure that ApoE4 x sTREM2 interactions were not driven by baseline differences in primary AD pathology (i.e. Aβ_1–42_, p-tau_181_), sTREM2 or any of the demographic variables. To account for multiple testing in our primary analysis using two different cognitive endpoints (i.e. ADNI-MEM & ADAS13), we applied a Bonferroni-corrected alpha-threshold of 0.025 (i.e. accounting for 2 tests). For all significant ApoE4 x sTREM2 interaction effects, we further computed effect size estimates (i.e. Cohen’s f^2^) which are interpreted as follows: 0.1 = small effect, 0.25 = medium effect, 0.4 = large effect. Note, that interaction effects were plotted using scores of the dependent variables that were residualized for CSF Aβ_1–42_ and p-tau_181_, in order to illustrate sTREM2 effects on the association between ApoE4 vs. cognitive and hippocampal volume changes independent of primary AD markers. As additional exploratory analyses, we re-ran the above described linear models this time testing whether a higher sTREM2/p-tau_181_ ratio consistently moderated the effect of ApoE4 on cognitive decline and neurodegeneration. Theses exploratory analyses were motivated by our previous work, showing that a higher sTREM2/p-tau_181_ ratio is associated with delayed conversion to AD dementia in amyloid-positive individuals [34]. All statistical analyses were conducted in R (Version 3.6.1).

## Results

Sample demographics, biomarker and cognitive data are shown in Table 1.

**Table 1:** Table 1: Sample characteristics

ADNI Sample (***N*** = 708)	CN (***n*** = 221)	MCI (***n*** = 414)	AD (***n*** = 73)	***p***-value
Age (M/SD)	74.25 (6.08)^b^	71.82 (7.45)^a,c^	74.17 (8.37)^b^	< 0.001
Gender (male/female))	115/106	244/170	38/35	0.190
Education (M/SD)	16.36 (2.73)^c^	16.14 (2.74)^c^	15.18 (3.06)^a,b^	0.007
Follow-up in years (M/SD)	4.89 (2.5)^b,c^	4.24 (1.81)^a,c^	2.15 (0.44)^a,b^	< 0.001
ApoE4-status (pos./neg.)	55/166	203/211	60/13	< 0.001
ApoE-alleles (22/23/33/24/34/44)	0/30/136/1/50/4	0/28/183/6/148/49	0/0/13/2/36/22	< 0.001
Amyloid-status (pos./neg.)	72/148	245/169	73/0	< 0.001
CSF-Aβ_1–42_ (M/SD)	1243.25 (421.93)^b,c^	976.67 (444.52)^a,c^	545.03 (159.15)=	< 0.001
CSF-p-tau_181_ (M/SD)	21.97 (9.24)^b,c^	27.55 (14.27)^a,c^	36.77 (14.33)^a,b^	< 0.001
CSF sTREM2 (M/SD)	4258.60 (2183.71)	4094.56 (2104.86)	4370.51 (2194.07)	0.465
ADNI-MEM (M/SD)	1.07 (0.58)^b,c^	0.23 (0.67)^a,c^	−0.84 (0.50)^a,b^	< 0.001
ADAS13 (M/SD)	9.08 (4.33)^b,c^	15.80 (6.61)^a,c^	28.82 (6.88)^a,b^	< 0.001
MMSE (M/SD)	29.11 (1.14)^b,c^	27.80 (1.78)^a,c^	23.12 (1.89)^a,b^	< 0.001

*CN* Cognitively Normal, *MCI* Mild Cognitive Impairment, *MMSE* Mini-Mental State Exam, *ADNI-MEM* Alzheimer’s disease Neuroimaging Initiative - memory composite, ^a^ = sig. Different from CN, ^b^ = sig. Different from MCI, ^c^ = sig. Different from Dementia

### ApoE4-carriage is associated with more abnormal AD biomarkers and faster cognitive decline but not with changed CSF sTREM2 levels

**Fig. 1:**
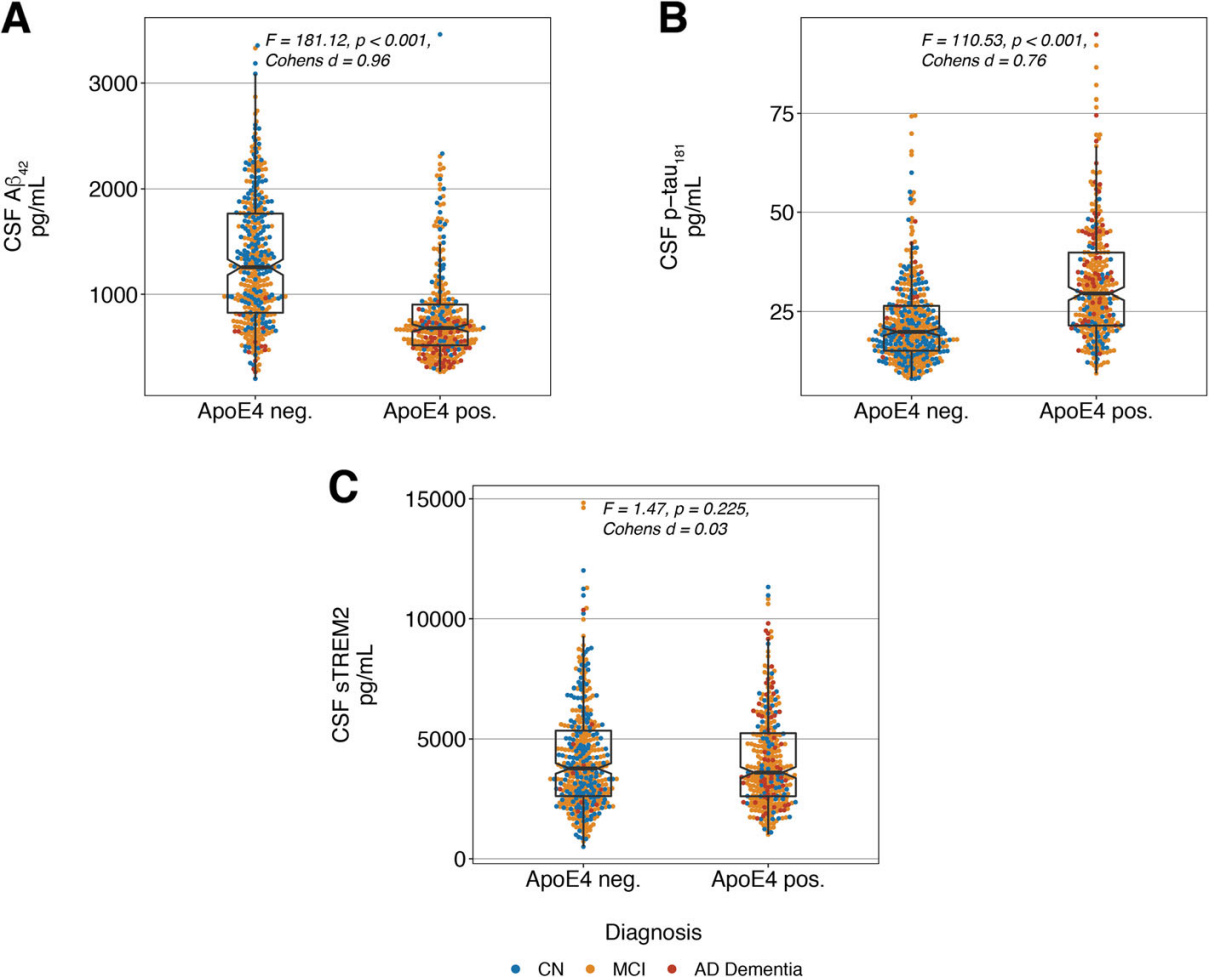
ApoE4 is associated with abnormal CSF Aβ_1–42_ and p-tau_181_ but not with sTREM2. Associations between ApoE4-status, baseline AD biomarkers ((**a**) Aβ_1–42_, (**b**) p-tau_181_) and (**c**) baseline sTREM2. F- and *p*-values were determined using ANCOVAs controlling for age, gender, education and diagnosis

**Fig. 2:**
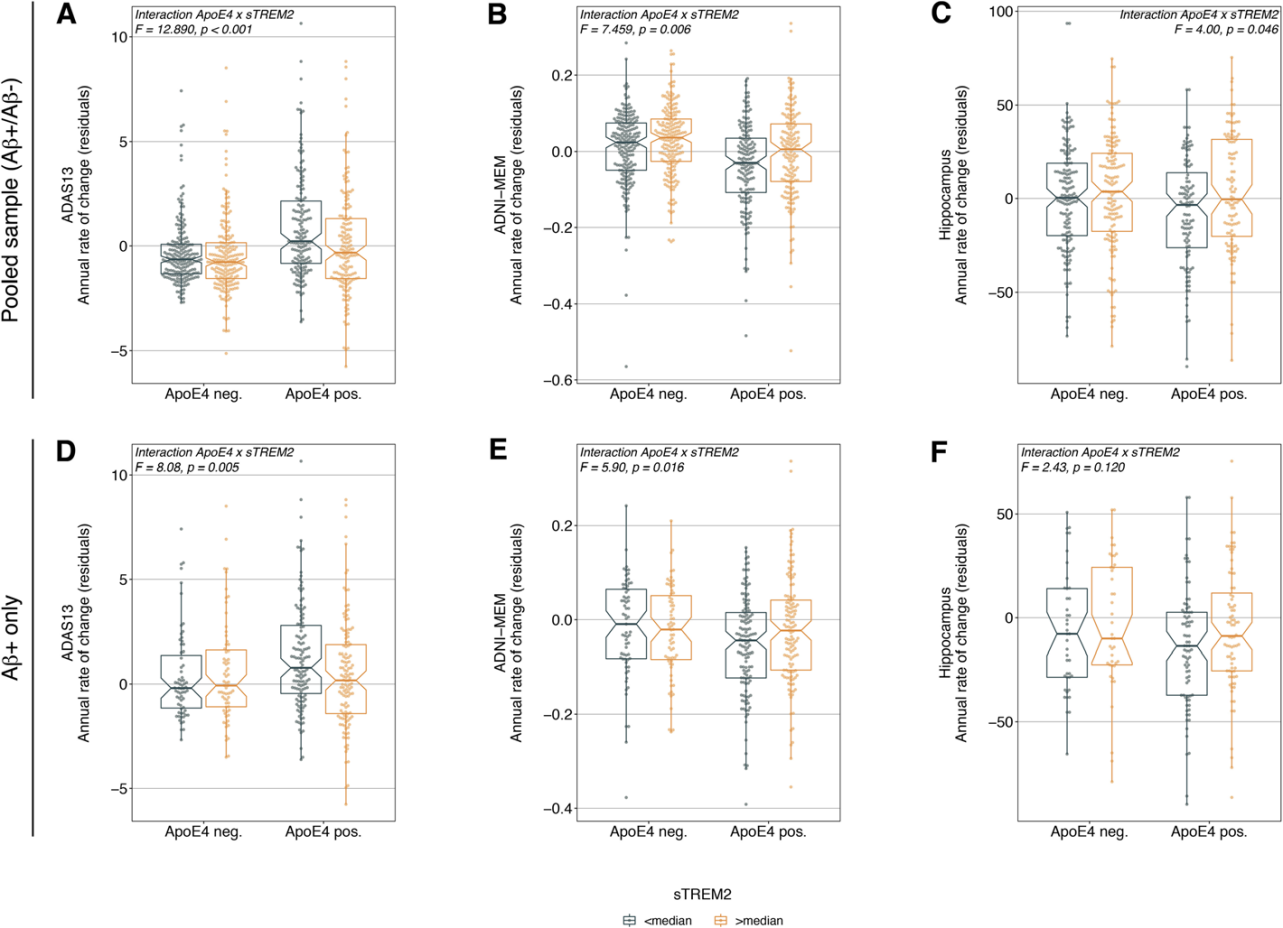
sTREM2 attenuates ApoE4 effects on cognitive decline and neurodegeneration. Panels **a-c** illustrate the interaction effect of sTREM2 on longitudinal ApoE4-related changes in global cognition (**a**), memory (**b**) and hippocampal volume changes (**c**) in the pooled Aβ+/Aβ- sample. For illustrational purposes, the sTREM2 levels are split at the median for ApoE4 negative and ApoE4 positive subjects. Statistics were, however, computed using continuous sTREM2 measures. In order to illustrate the sTREM2 effects on cognition and hippocampal volume changes independent of primary AD pathology y-axis are residualized for CSF Aβ_1–42_ and p-tau_181_. Panels **d-f** illustrate the same interaction effects of sTREM2 on longitudinal ApoE4-related changes restricted to Aβ + subjects

First, we assessed the association between ApoE4-carriage, baseline CSF biomarkers, rates of cognitive decline and hippocampal atrophy. We found an association between ApoE4-carriage and both decreased baseline CSF-Aβ_1–42_ (F = 182.12, *p* < 0.001, Cohens D = 0.96, Fig. 1a, Supplementary Figure 1A for a stratification by diagnosis) and higher baseline p-tau_181_ levels (F = 110.53, *p* < 0.001, Cohens D = 0.76, Fig. 1b, Supplementary Figure 1B stratified by diagnosis), using ANCOVAs controlled for age, gender, education and diagnosis. ApoE4-carriage was, however, not associated with baseline CSF sTREM2 levels (F = 1.47, *p* = 0.225, Cohens D = 0.03, Fig. 1c, ANCOVA controlled for age, gender, education and diagnosis, Supplementary Figure 1C stratified by diagnosis), consistent with previous reports [43]. For cognition, we found an association between ApoE4-carriage and faster decline in both ADNI-MEM (F (1,693) = 64.61, *p* < 0.001, Cohens D = 0.51, Fig. 2a) and ADAS13 (F (1,690) = 91.10, *p* < 0.001, Cohens D = 0.58, Fig. 2b) controlling for age, gender, education, diagnosis, follow-up duration and baseline cognition (i.e. ADNI-MEM or ADAS13 respectively). In a similar vein, ApoE4-carriage was associated with faster hippocampal atrophy, controlling for age, gender, education, diagnosis baseline hippocampal and intracranial volume (F = 12.39, *p* < 0.001, Cohens D = 0.30). Together, these results indicate that ApoE4-carriage is - independently of clinical diagnostic status - associated with AD-typical CSF amyloid and tau levels, as well as cognitive decline and neurodegeneration in the current ADNI sample. However, ApoE4-carriage is not associated with elevated CSF sTREM2 levels.

### ApoE4-effects on cognitive decline are attenuated at higher sTREM2 levels

We next tested our main hypothesis that higher sTREM2 levels are associated with attenuated effects of ApoE4-carriage on future cognitive decline. For our main analysis, we found a significant interaction between sTREM2 and ApoE4-carriage on the change rates in ADAS13 (F = 12.89, *p* < 0.001, Cohens *f*^*2*^ = 0.137, Fig. 2a) and ADNI-MEM (F = 7.459, *p* = 0.006, Cohens *f*^*2*^ = 0.104, Fig. 2b) controlling for main effects of CSF p-tau_181_, Aβ_42_, sTREM2, ApoE4 plus age, gender, education, diagnosis, follow-up duration, and baseline cognition (i.e. ADNI-MEM or ADAS13). Note that changes in ADAS13 and ADNI-MEM as shown in Fig. 2 have been split at the median and residualized for CSF Aβ and p-tau_181_ for illustrational purposes only, while statistics were derived using continuous and raw ADAS13 or ADNI-MEM scores. As shown in Fig. 2a&b, higher sTREM2 levels at baseline were associated with an attenuated effect of ApoE4-carriage on future cognitive decline (for equivalent plots using non-residualized cognitive change scores see supplementary Figure 2). Results remained significant after applying a Bonferroni-corrected alpha-threshold of 0.025, accounting for two independent cognitive endpoints. Congruent interaction effects were found when testing the sTREM2/p-tau_181_ x ApoE4 interaction, controlling for main effects of sTREM2, ApoE4, p-tau_181_, age, gender, education, diagnosis, follow-up duration, as well as baseline cognition (ADAS13: F = 9.39, *p* = 0.002, Cohens *f*^*2*^ *=* 0.117; ADNI-MEM: F = 14.54, *p* < 0.001, Cohens *f*^*2*^ *= 0.145*).

In an exploratory step, we further assessed whether sTREM2 x ApoE4 interactions on cognitive decline were driven by the presence of abnormal amyloid levels. To address this, we additionally restricted the models to Aβ + subjects, where we found congruent results with our main analyses (ADAS13: F = 8.08, *p* = 0.005, Cohens *f*^*2*^ = 0.147, Fig. 2d; ADNI-MEM: F = 5.90, *p* = 0.016, Cohens *f*^*2*^ = 0.125, Fig. 2e). To further determine whether our findings were driven by clinical diagnosis, we repeated the analyses stratified by diagnostic group and found sTREM2 x ApoE4 interaction effects to be in line with our main analyses in MCI (ADNI-MEM: F = 5.00, *p* = 0.026, Cohens *f*^*2*^ = 0.112; ADAS13: F = 10.41, *p* = 0.001, Cohens *f*^*2*^ *=* 0.162). In CN and AD, we found trend level sTREM2 x ApoE4 interactions for rates of change in ADAS13 (CN: F = 3.78, *p* = 0.053, Cohens *f*^*2*^ = 0.135; AD: F = 3.23, *p* = 0.07, Cohens *f*^*2*^ = 0.230), but no effects on rates of change in ADNI-MEM (CN: *p* = 0.95, AD: *p* = 0.22). Collectively, these analyses suggest that higher sTREM2 levels are associated with attenuated effects of ApoE4-carriage on future cognitive decline.

### High sTREM2 levels attenuate ApoE4-effects on neurodegeneration

Lastly, we determined whether higher sTREM2 levels attenuate the effect of ApoE4-carriage on neurodegeneration (i.e. annual hippocampal volume change rates). Here, we found a significant sTREM2 x ApoE4 interaction on annual hippocampal volume changes (F = 4.00, *p* = 0.046, Cohens *f*^*2*^ = 0.089, Fig. 2c, model controlled for main effects of ApoE4 and sTREM2, baseline levels of p-tau_181_, Aβ_1–42_, age, gender, education, diagnosis, follow-up duration, baseline hippocampal and intracranial volume). Figure 2c illustrates that ApoE4-carriers with higher sTREM2 levels at baseline showed slower future hippocampal atrophy. The sTREM2 x ApoE4 interaction remained significant when tested in MCI (F = 7.72, *p* = 0.006 Cohens *f*^*2*^ = 0.147), but was non-significant when tested in CN (*p* = 0.35), AD (*p* = 0.20) or Aβ + only (F = 2.43, *p* = 0.120, Fig. 2f). A congruent effect on hippocampal change rates was found when testing the sTREM2/p-tau_181_ x ApoE4 interaction (F = 9.78, *p* = 0.002, Cohens *f*^*2*^ = 0.134, i.e. using the same covariates as the previous model) in the entire Aβ+/Aβ- sample. Faster hippocampal atrophy rates were associated with faster decline in ADNI-MEM (β = 0.34, T = 8.37, *p* < 0.001) and ADAS13 (β = − 0.21, T = -5.32, *p* < 0.001), as shown by linear regression controlling for age, gender, education and diagnosis. Together, these findings indicate that higher sTREM2 levels are associated with slower hippocampal atrophy in ApoE4-carriers, thereby potentially attenuating cognitive changes.

## Discussion

Here we show that higher sTREM2 levels are associated with attenuated ApoE4-effects on future global cognitive and memory decline as well as AD-typical hippocampal neurodegeneration in a large sample of cognitively normal to AD dementia subjects. These effects were statistically independent of baseline Aβ-levels or diagnostic status. Importantly, CSF sTREM2 levels were – in contrast to CSF markers of Aβ and tau pathology – not associated with ApoE4-carriage, confirming previous evidence that CSF sTREM2 concentrations [43] or PET-assessed microglial activation [44] are per se unrelated to ApoE4-associated genetic AD risk. Given that CSF sTREM2 levels most likely reflect the expression levels of signaling competent TREM2 on activated microglia [27, 30], our findings indicate that a higher TREM2-related neuroimmune response may be protective against the clinical and neurodegenerative consequences of ApoE4-carriage, the strongest genetic risk factor for sporadic AD.

Consistent with previous studies showing associations between ApoE4 and more abnormal AD biomarkers, we found that ApoE4-carriers had overall stronger abnormal changes in CSF markers of Aβ_1–42_ and p-tau_181_ at baseline, supporting the role of ApoE4 as a major AD risk factor [45, 46]. In addition, ApoE4 carriers showed faster rates of cognitive decline and neurodegeneration, suggesting greater risk for developing AD-related cognitive impairment in ApoE4 positive individuals of the ADNI cohort. However, ApoE4-related risk for accelerated cognitive decline and neurodegeneration was attenuated at higher sTREM2 levels, independent of diagnosis or baseline levels of AD pathology, suggesting that relatively high microglial sTREM2 may compensate the ApoE4-related increase in the risk to develop cognitive decline. Similarly, we showed previously in biomarker defined AD patients (i.e. patients with abnormal amyloid and tau levels), that higher sTREM2-levels were associated with delayed cognitive decline and clinical progression [34]. The current results critically extend these previous findings [34], suggesting that a TREM2-related neuroimmune response may attenuate the effects of the strongest known genetic risk factor for developing sporadic AD. These findings are of high clinical relevance since TREM2 signaling is potentially modifiable, as shown recently with TREM2-agonistic antibodies [26].

For neurodegeneration, we found that a higher TREM2-related microglia response attenuates the effect of ApoE4-carriage on atrophy of the hippocampus. Again, these results were independent of primary AD pathology markers (i.e. CSF Aβ_1–42_, p-tau_181_). We and others have previously reported reduced grey matter atrophy at higher CSF sTREM2 levels at a given level of primary AD pathology in AD patients [47]. A study combining TSPO-PET with structural MRI in AD patients could show that higher TSPO-PET was associated with higher grey matter volume, favoring a neuroprotective effect of microglial activation [44]. The latter study further reported that higher TSPO-related grey matter volume was associated with better cognition, suggesting that higher grey matter volume indeed reflects preserved neuronal integrity, rather than inflammation-mediated edema [44]. In the context of the current findings, it is thus possible that a TREM2-related neuroimmune response in ApoE4-carriers attenuates either an amyloid-induced deposition of tau pathology itself or the downstream consequences of tau that ultimately lead to neurodegeneration and cognitive decline [48]. Together, these previous findings suggest that protective effects of TREM2 may be observed on different levels, e.g. on primary AD pathology itself or on its’ consequences. It will thus be important for future studies to address these questions on different levels, e.g. by disentangling the molecular interactions between microglia and primary AD pathology and by combining PET-imaging of primary AD pathologies and microglial activation with MRI and CSF sTREM2 assessments to better understand the interplay of microglial activation with in vivo markers of AD.

Whether a TREM2-related microglial response to AD pathology is adaptive or detrimental for the development of AD has been intensely debated with conflicting results from preclinical studies [49]. Supporting a protective role of TREM2, preclinical studies have reported that TREM2-related microglial activation supports phagocytosis of Aβ [20], limits amyloid [22] and tau seeding [24] as well as tau hyperphosphorylation [23]. Supporting this, experimentally increasing sTREM2 levels in 5xFAD mice enhances microglial proliferation and phagocytosis of Aβ and limits Aβ neurotoxicity [50]. In agreement with this finding, we showed recently in humans and APP mice that elevated sTREM2 levels are associated with slower PET-assessed Aβ accumulation [51]. In a similar vein, microglia modulation via TREM2 agonistic antibodies has been shown to improve Aβ phagocytosis in transgenic AD-mouse models [26]. A protective effect of TREM2 is further suggested by post-mortem assessments, showing that TREM2 function promotes microglial clustering and Aβ plaque encapsulation that is associated with attenuated accumulation of tau pathology [24]. Similarly, studies in pure tauopathy mice (i.e. P301S) have shown that TREM2 overexpression ameliorates tau hyperphosphorylation, neurodegeneration and cognitive decline, by suppressing neuroinflammation induced tau kinases [23, 25]. In contrast, others have argued that TREM2 exacerbates both amyloid and tau pathology in AD mice [52]. Conflicting findings from animal models may stem from various factors, including the use of different disease models or the investigation of TREM2 at different disease stages or severity levels of primary AD pathology [49]. Despite conflicting pre-clinical findings on a protective vs. detrimental role of TREM2, our current and previous findings in humans [34, 51] favor a protective role of TREM2 against the development of AD pathology, AD-related cognitive decline and neurodegeneration.

Several caveats should be considered when interpreting the current results. First, while the link between ApoE4 and AD pathology is clearly established [46], there exist mixed and partly inconsistent reports on whether ApoE4-carriage is indeed associated with cognition and cognitive changes, depending on age and selection of the study population [8, 9, 11, 53–55]. However, results from several previous studies indicate that ApoE4-associated cognitive changes are pronounced specifically at older age [9–11]. Thus, we believe that the current sample with a mean age of ~ 73 years is well-suited to study ApoE4 effects on cognitive changes. Second, previous studies have reported increases of sTREM2 in AD, specifically in Aβ and tau-positive subjects [32, 34]. Hence, the absence of group-specific (i.e. CN, MCI, AD dementia) sTREM2 increases in the current study is potentially driven by our study design including subjects with mixed levels of both Aβ and tau pathology across diagnostic groups. Third, CSF sTREM2 levels are only an indirect measure of TREM2 signaling [30], hence direct conclusions on the level of microglia activation cannot be drawn unless our findings are replicated with PET imaging of microglial activation or by TREM2 antibody mediated microglial modulation [26]. Fourth, microglial activation in neurodegenerative diseases is highly complex and requires an interplay of multiple signaling pathways besides TREM2 [56], which were not assessed in the current study. Future studies will thus need to investigate further how the complex molecular signature of activated microglia may modulate AD progression. Fifth, the current study is observational in nature, hence we emphasize that causative conclusions are not necessarily implied by our findings.

## Conclusions

In conclusion, we show that higher sTREM2 levels attenuate the effect of ApoE4-carriage, i.e. the strongest genetic risk factor for sporadic AD, on future cognitive decline and neurodegeneration. These findings have important clinical implications, since TREM2 may reduce the overall risk to develop AD. Together, our findings suggest that targeting TREM2 could serve as a promising strategy for treating and preventing AD.

## Supplementary information

**Additional file 1.**

## Data Availability

The dataset supporting the conclusions of this manuscript is available at the ADNI website (http://adni.loni.usc.edu/). Data used in preparation of this article were obtained from the Alzheimer’s Disease Neuroimaging Initiative (ADNI) database (adni.loni.usc.edu). As such, the investigators within the ADNI contributed to the design and implementation of ADNI and/or provided data but did not participate in analysis or writing of this report. A complete listing of ADNI investigators can be found in the appendix (“ADNI_coinvestigators.docx”).

## Abbreviations

Aβ: Amyloid-beta
AD: Alzheimer’s disease
ADAS13: Alzheimer’s disease assessment scale 13
ADAM10/17: A Disintegrin and metalloproteinase domain-containing protein 10/17
ADNI: Alzheimer’s disease neuroimaging initiative
ANCOVA: Analysis of Covariance
ApoE4: Apolipoprotein 4
CDR: Clinical dementia rating
CSF: Cerebrospinal fluid
DAP12: DNAX-activating protein of 12 kDA
ELISA: Enzyme-linked immunosorbent assay
GWAS: Genome wide association studies
MMSE: Mini-Mental State Examination
MPRAGE: Magentization prepared rapid gradient echo
MRI: Magnetic Resonance Imaging
PET: Positron Emission Tomography
p-tau: Phosphorylated tau
TREM2: Triggering receptor expressed on myeloid cells 2
TSPO: Translocator Protein
sTREM2: Soluble TREM2

## References

[1] Belloy ME, Napolioni V, Greicius MD (2019). A quarter century of APOE and Alzheimer's disease: Progress to date and the path forward. Neuron.

[2] Qian J, Wolters FJ, Beiser A, Haan M, Ikram MA, Karlawish J, Langbaum JB, Neuhaus JM, Reiman EM, Roberts JS (2017). APOE-related risk of mild cognitive impairment and dementia for prevention trials: an analysis of four cohorts. PLoS Med.

[3] Blacker D, Haines JL, Rodes L, Terwedow H, Go RC, Harrell LE, Perry RT, Bassett SS, Chase G, Meyers D (1997). ApoE-4 and age at onset of Alzheimer's disease: the NIMH genetics initiative. Neurology.

[4] Lautner R, Insel PS, Skillback T, Olsson B, Landen M, Frisoni GB, Herukka SK, Hampel H, Wallin A, Minthon L (2017). Preclinical effects of APOE epsilon4 on cerebrospinal fluid Abeta42 concentrations. Alzheimers Res Ther.

[5] Gonneaud J, Arenaza-Urquijo EM, Fouquet M, Perrotin A, Fradin S, de La Sayette V, Eustache F, Chetelat G (2016). Relative effect of APOE epsilon4 on neuroimaging biomarker changes across the lifespan. Neurology.

[6] Fleisher AS, Chen K, Liu X, Ayutyanont N, Roontiva A, Thiyyagura P, Protas H, Joshi AD, Sabbagh M, Sadowsky CH (2013). Apolipoprotein E epsilon4 and age effects on florbetapir positron emission tomography in healthy aging and Alzheimer disease. Neurobiol Aging.

[7] Therriault J, Benedet AL, Pascoal TA, Mathotaarachchi S, Chamoun M, Savard M, Thomas E, Kang MS, Lussier F, Tissot C (2020). Association of Apolipoprotein E epsilon4 with medial temporal tau independent of amyloid-beta. JAMA Neurol.

[8] Rawle MJ, Davis D, Bendayan R, Wong A, Kuh D, Richards M (2018). Apolipoprotein-E (Apoe) epsilon4 and cognitive decline over the adult life course. Transl Psychiatry.

[9] Caselli RJ, Dueck AC, Osborne D, Sabbagh MN, Connor DJ, Ahern GL, Baxter LC, Rapcsak SZ, Shi J, Woodruff BK (2009). Longitudinal modeling of age-related memory decline and the APOE epsilon4 effect. N Engl J Med.

[10] Christensen H, Batterham PJ, Mackinnon AJ, Jorm AF, Mack HA, Mather KA, Anstey KJ, Sachdev PS, Easteal S (2008). The association of APOE genotype and cognitive decline in interaction with risk factors in a 65-69 year old community sample. BMC Geriatr.

[11] Makkar SR, Lipnicki DM, Crawford JD, Kochan NA, Castro-Costa E, Lima-Costa MF, Diniz BS, Brayne C, Stephan B, Matthews F, et al. APOE epsilon4 and the influence of sex, age, vascular risk factors, and ethnicity on cognitive decline. J Gerontol A Biol Sci Med Sci. 2020;75(10):1863-73. 10.1093/gerona/glaa116. 10.1093/gerona/glaa116 PMC7518559 32396611

[12] Cacciaglia R, Molinuevo JL, Falcon C, Brugulat-Serrat A, Sanchez-Benavides G, Gramunt N, Esteller M, Moran S, Minguillon C, Fauria K (2018). Effects of APOE-epsilon4 allele load on brain morphology in a cohort of middle-aged healthy individuals with enriched genetic risk for Alzheimer's disease. Alzheimers Dement.

[13] International Genomics of Alzheimer's Disease C (2015). Convergent genetic and expression data implicate immunity in Alzheimer's disease. Alzheimers Dement.

[14] Jansen IE, Savage JE, Watanabe K, Bryois J, Williams DM, Steinberg S, Sealock J, Karlsson IK, Hagg S, Athanasiu L (2019). Genome-wide meta-analysis identifies new loci and functional pathways influencing Alzheimer's disease risk. Nat Genet.

[15] Kunkle BW, Grenier-Boley B, Sims R, Bis JC, Damotte V, Naj AC, Boland A, Vronskaya M, van der Lee SJ, Amlie-Wolf A (2019). Genetic meta-analysis of diagnosed Alzheimer's disease identifies new risk loci and implicates Abeta, tau, immunity and lipid processing. Nat Genet.

[16] Jonsson T, Stefansson H, Steinberg S, Jonsdottir I, Jonsson PV, Snaedal J, Bjornsson S, Huttenlocher J, Levey AI, Lah JJ (2013). Variant of TREM2 associated with the risk of Alzheimer's disease. N Engl J Med.

[17] Mazaheri F, Snaidero N, Kleinberger G, Madore C, Daria A, Werner G, Krasemann S, Capell A, Trumbach D, Wurst W (2017). TREM2 deficiency impairs chemotaxis and microglial responses to neuronal injury. EMBO Rep.

[18] Takahashi K, Rochford CD, Neumann H (2005). Clearance of apoptotic neurons without inflammation by microglial triggering receptor expressed on myeloid cells-2. J Exp Med.

[19] Kleinberger G, Yamanishi Y, Suarez-Calvet M, Czirr E, Lohmann E, Cuyvers E, Struyfs H, Pettkus N, Wenninger-Weinzierl A, Mazaheri F (2014). TREM2 mutations implicated in neurodegeneration impair cell surface transport and phagocytosis. Sci Transl Med.

[20] Yeh FL, Wang Y, Tom I, Gonzalez LC, Sheng M. TREM2 binds to Apolipoproteins, including APOE and CLU/APOJ, and thereby facilitates uptake of amyloid-Beta by microglia. Neuron. 25;75(10):1863-1873. 10.1093/gerona/glaa116. 10.1016/j.neuron.2016.06.015 27477018

[21] Wang Y, Cella M, Mallinson K, Ulrich JD, Young KL, Robinette ML, Gilfillan S, Krishnan GM, Sudhakar S, Zinselmeyer BH (2015). TREM2 lipid sensing sustains the microglial response in an Alzheimer's disease model. Cell.

[22] Parhizkar S, Arzberger T, Brendel M, Kleinberger G, Deussing M, Focke C, Nuscher B, Xiong M, Ghasemigharagoz A, Katzmarski N (2019). Loss of TREM2 function increases amyloid seeding but reduces plaque-associated ApoE. Nat Neurosci.

[23] Jiang T, Zhang YD, Gao Q, Ou Z, Gong PY, Shi JQ, Wu L, Zhou JS (2018). TREM2 ameliorates neuronal tau pathology through suppression of microglial inflammatory response. Inflammation.

[24] Leyns CEG, Gratuze M, Narasimhan S, Jain N, Koscal LJ, Jiang H, Manis M, Colonna M, Lee VMY, Ulrich JD, Holtzman DM (2019). TREM2 function impedes tau seeding in neuritic plaques. Nat Neurosci.

[25] Jiang T, Zhang YD, Chen Q, Gao Q, Zhu XC, Zhou JS, Shi JQ, Lu H, Tan L, Yu JT (2016). TREM2 modifies microglial phenotype and provides neuroprotection in P301S tau transgenic mice. Neuropharmacology.

[26] Schlepckow K, Monroe KM, Kleinberger G, Cantuti-Castelvetri L, Parhizkar S, Xia D, Willem M, Werner G, Pettkus N, Brunner B (2020). Enhancing protective microglial activities with a dual function TREM2 antibody to the stalk region. EMBO Mol Med.

[27] Suarez-Calvet M, Kleinberger G, Araque Caballero MA, Brendel M, Rominger A, Alcolea D, Fortea J, Lleo A, Blesa R, Gispert JD (2016). sTREM2 cerebrospinal fluid levels are a potential biomarker for microglia activity in early-stage Alzheimer's disease and associate with neuronal injury markers. EMBO Mol Med.

[28] Heslegrave A, Heywood W, Paterson R, Magdalinou N, Svensson J, Johansson P, Ohrfelt A, Blennow K, Hardy J, Schott J (2016). Increased cerebrospinal fluid soluble TREM2 concentration in Alzheimer's disease. Mol Neurodegener.

[29] Piccio L, Deming Y, Del-Aguila JL, Ghezzi L, Holtzman DM, Fagan AM, Fenoglio C, Galimberti D, Borroni B, Cruchaga C (2016). Cerebrospinal fluid soluble TREM2 is higher in Alzheimer disease and associated with mutation status. Acta Neuropathol.

[30] Brendel M, Kleinberger G, Probst F, Jaworska A, Overhoff F, Blume T, Albert NL, Carlsen J, Lindner S, Gildehaus FJ (2017). Increase of TREM2 during aging of an Alzheimer's disease mouse model is paralleled by microglial activation and amyloidosis. Front Aging Neurosci.

[31] Kleinberger G, Brendel M, Mracsko E, Wefers B, Groeneweg L, Xiang X, Focke C, Deussing M, Suarez-Calvet M, Mazaheri F (2017). The FTD-like syndrome causing TREM2 T66M mutation impairs microglia function, brain perfusion, and glucose metabolism. EMBO J.

[32] Suarez-Calvet M, Morenas-Rodriguez E, Kleinberger G, Schlepckow K, Araque Caballero MA, Franzmeier N, Capell A, Fellerer K, Nuscher B, Eren E (2019). Early increase of CSF sTREM2 in Alzheimer's disease is associated with tau related-neurodegeneration but not with amyloid-beta pathology. Mol Neurodegener.

[33] Suarez-Calvet M, Araque Caballero MA, Kleinberger G, Bateman RJ, Fagan AM, Morris JC, Levin J, Danek A, Ewers M, Haass C, Dominantly Inherited Alzheimer N (2016). Sci Transl Med.

[34] Ewers M, Franzmeier N, Suarez-Calvet M, Morenas-Rodriguez E, Caballero MAA, Kleinberger G, Piccio L, Cruchaga C, Deming Y, Dichgans M, et al. Increased soluble TREM2 in cerebrospinal fluid is associated with reduced cognitive and clinical decline in Alzheimer's disease. Sci Transl Med. 2019;11(507):eaav6221. 10.1126/scitranslmed.aav6221.. 10.1126/scitranslmed.aav6221 PMC7050285 31462511

[35] Petersen RC, Aisen PS, Beckett LA, Donohue MC, Gamst AC, Harvey DJ, Jack CR, Jagust WJ, Shaw LM, Toga AW (2010). Alzheimer's disease neuroimaging initiative (ADNI): clinical characterization. Neurology.

[36] Hansson O, Seibyl J, Stomrud E, Zetterberg H, Trojanowski JQ, Bittner T, Lifke V, Corradini V, Eichenlaub U, Batrla R (2018). CSF biomarkers of Alzheimer's disease concord with amyloid-beta PET and predict clinical progression: a study of fully automated immunoassays in BioFINDER and ADNI cohorts. Alzheimers Dement.

[37] Tosun D, Schuff N, Shaw LM, Trojanowski JQ, Weiner MW, Alzheimer's disease NeuroImaging I (2011). Relationship between CSF biomarkers of Alzheimer's disease and rates of regional cortical thinning in ADNI data. J Alzheimers Dis.

[38] Jack CR, Barnes J, Bernstein MA, Borowski BJ, Brewer J, Clegg S, Dale AM, Carmichael O, Ching C, DeCarli C (2015). Magnetic resonance imaging in Alzheimer's disease neuroimaging initiative 2. Alzheimers Dement.

[39] Crane PK, Carle A, Gibbons LE, Insel P, Mackin RS, Gross A, Jones RN, Mukherjee S, Curtis SM, Harvey D (2012). Development and assessment of a composite score for memory in the Alzheimer's disease neuroimaging initiative (ADNI). Brain Imaging Behav.

[40] Mohs RC, Knopman D, Petersen RC, Ferris SH, Ernesto C, Grundman M, Sano M, Bieliauskas L, Geldmacher D, Clark C, Thal LJ (1997). Development of cognitive instruments for use in clinical trials of antidementia drugs: additions to the Alzheimer's Disease Assessment Scale that broaden its scope. The Alzheimer's Disease Cooperative Study. Alzheimer Dis Assoc Disord.

[41] Rosen WG, Mohs RC, Davis KL (1984). A new rating scale for Alzheimer's disease. Am J Psychiatry.

[42] Preische O, Schultz SA, Apel A, Kuhle J, Kaeser SA, Barro C, Graber S, Kuder-Buletta E, LaFougere C, Laske C (2019). Serum neurofilament dynamics predicts neurodegeneration and clinical progression in presymptomatic Alzheimer's disease. Nat Med.

[43] Gispert JD, Monte GC, Suarez-Calvet M, Falcon C, Tucholka A, Rojas S, Rami L, Sanchez-Valle R, Llado A, Kleinberger G (2017). The APOE epsilon4 genotype modulates CSF YKL-40 levels and their structural brain correlates in the continuum of Alzheimer's disease but not those of sTREM2. Alzheimers Dement (Amst).

[44] Hamelin L, Lagarde J, Dorothee G, Leroy C, Labit M, Comley RA, de Souza LC, Corne H, Dauphinot L, Bertoux M (2016). Early and protective microglial activation in Alzheimer's disease: a prospective study using 18F-DPA-714 PET imaging. Brain.

[45] Risacher SL, Kim S, Nho K, Foroud T, Shen L, Petersen RC, Jack CR, Beckett LA, Aisen PS, Koeppe RA (2015). APOE effect on Alzheimer's disease biomarkers in older adults with significant memory concern. Alzheimers Dement.

[46] Liu Y, Yu JT, Wang HF, Han PR, Tan CC, Wang C, Meng XF, Risacher SL, Saykin AJ, Tan L (2015). APOE genotype and neuroimaging markers of Alzheimer's disease: systematic review and meta-analysis. J Neurol Neurosurg Psychiatry.

[47] Gispert JD, Suarez-Calvet M, Monte GC, Tucholka A, Falcon C, Rojas S, Rami L, Sanchez-Valle R, Llado A, Kleinberger G (2016). Cerebrospinal fluid sTREM2 levels are associated with gray matter volume increases and reduced diffusivity in early Alzheimer's disease. Alzheimers Dement.

[48] La Joie R, Visani AV, Baker SL, Brown JA, Bourakova V, Cha J, Chaudhary K, Edwards L, Iaccarino L, Janabi M, et al. Prospective longitudinal atrophy in Alzheimer's disease correlates with the intensity and topography of baseline tau-PET. Sci Transl Med. 2020;12. 10.1126/scitranslmed.aau5732 PMC7035952 31894103

[49] Gratuze M, Leyns CEG, Holtzman DM (2018). New insights into the role of TREM2 in Alzheimer's disease. Mol Neurodegener.

[50] Zhong L, Xu Y, Zhuo R, Wang T, Wang K, Huang R, Wang D, Gao Y, Zhu Y, Sheng X (2019). Soluble TREM2 ameliorates pathological phenotypes by modulating microglial functions in an Alzheimer's disease model. Nat Commun.

[51] Ewers M, Biechele G, Suarez-Calvet M, Sacher C, Blume T, Morenas-Rodriguez E, Deming Y, Piccio L, Cruchaga C, Kleinberger G (2020). Higher CSF sTREM2 and microglia activation are associated with slower rates of beta-amyloid accumulation. EMBO Mol Med.

[52] Jay TR, Miller CM, Cheng PJ, Graham LC, Bemiller S, Broihier ML, Xu G, Margevicius D, Karlo JC, Sousa GL (2015). TREM2 deficiency eliminates TREM2+ inflammatory macrophages and ameliorates pathology in Alzheimer's disease mouse models. J Exp Med.

[53] Bunce D, Bielak AA, Anstey KJ, Cherbuin N, Batterham PJ, Easteal S (2014). APOE genotype and cognitive change in young, middle-aged, and older adults living in the community. J Gerontol A Biol Sci Med Sci.

[54] Growdon JH, Locascio JJ, Corkin S, Gomez-Isla T, Hyman BT (1996). Apolipoprotein E genotype does not influence rates of cognitive decline in Alzheimer's disease. Neurology.

[55] Jorm AF, Mather KA, Butterworth P, Anstey KJ, Christensen H, Easteal S (2007). APOE genotype and cognitive functioning in a large age-stratified population sample. Neuropsychology.

[56] Krasemann S, Madore C, Cialic R, Baufeld C, Calcagno N, El Fatimy R, Beckers L, O'Loughlin E, Xu Y, Fanek Z (2017). The TREM2-APOE pathway drives the transcriptional phenotype of dysfunctional microglia in neurodegenerative diseases. Immunity.

