# Supplementary material for: Higher CSF sTREM2 attenuates ApoE4-related risk for cognitive decline and neurodegeneration
Source: Mol Neurodegener. 2020 Oct 8;15:57. doi: 10.1186/s13024-020-00407-2 (PMC7545547; doi:10.1186/s13024-020-00407-2)
Supplement: Supplementary file 1 — Additional file 1. [file 13024_2020_407_MOESM1_ESM.docx]

*Supplementary Figure 1:
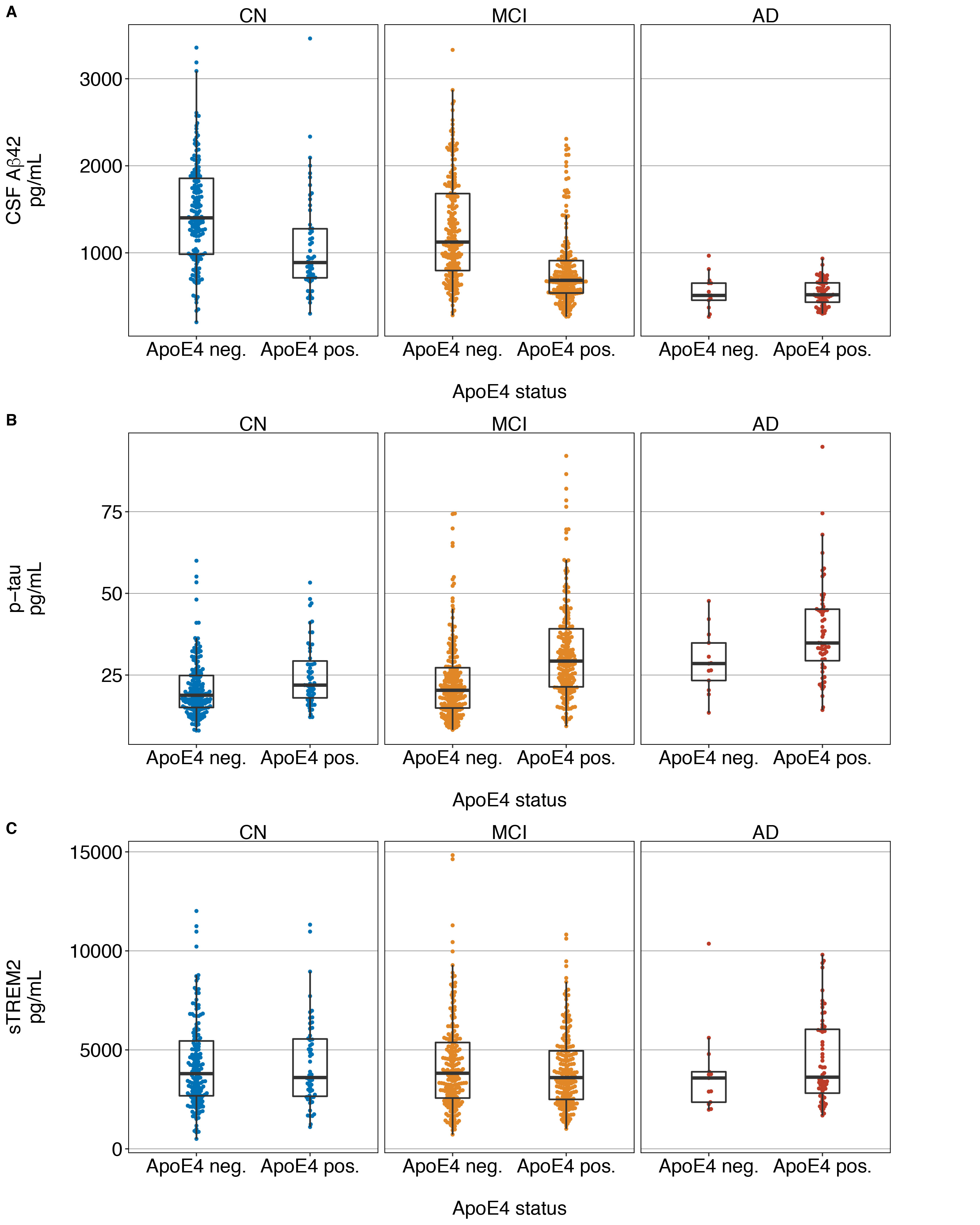
*

*Associations between ApoE4-status, baseline AD biomarkers ((A) Aβ_1-42_, (B) p-tau_181_) and (C) baseline sTREM2 stratified by diagnostic groups*

*Supplementary Figure 2:*

*
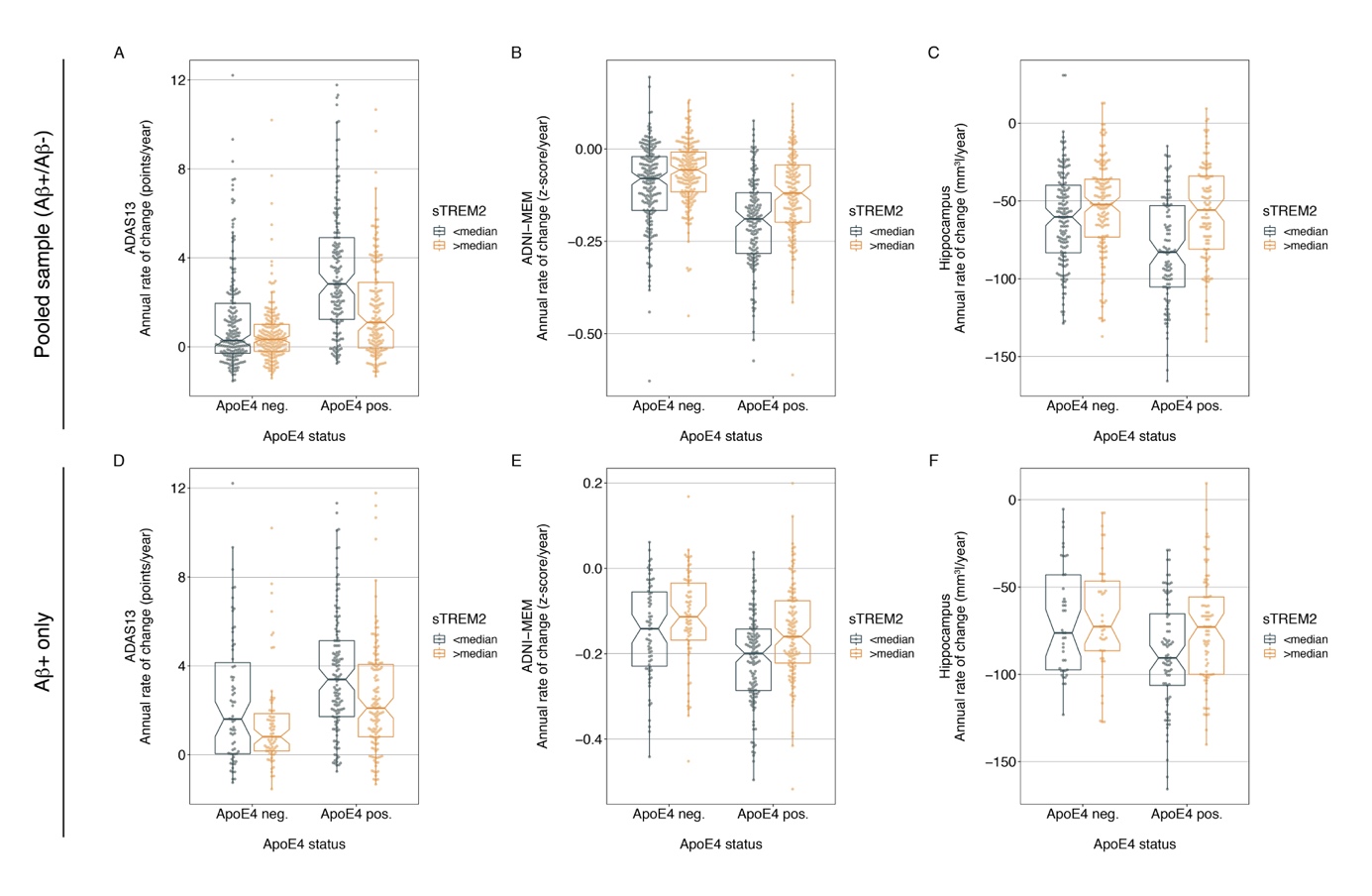
*

*Panels A-C illustrate the interaction effect of sTREM2 on longitudinal ApoE4-related changes in global cognition (A), memory (B) and hippocampal volume changes (C) in the pooled Aβ+/Aβ- sample using raw cognitive change rates. For illustrational purposes, the sTREM2 levels are split at the median for ApoE4 negative and ApoE4 positive subjects. Statistics were, however, computed using continuous sTREM2 measures. Panels D-F illustrate the same interaction effects of sTREM2 on longitudinal ApoE4-related changes restricted to Aβ+ subjects.*
